# The Remarkable Evolutionary Plasticity of Coronaviruses by Mutation and Recombination: Insights for the COVID-19 Pandemic and the Future Evolutionary Paths of SARS-CoV-2

**DOI:** 10.3390/v14010078

**Published:** 2022-01-02

**Authors:** Grigorios D. Amoutzias, Marios Nikolaidis, Eleni Tryfonopoulou, Katerina Chlichlia, Panayotis Markoulatos, Stephen G. Oliver

**Affiliations:** 1 Bioinformatics Laboratory, Department of Biochemistry and Biotechnology, University of Thessaly, 41500 Larissa, Greece; marionik23@gmail.com; 2 Laboratory of Molecular Immunology, Department of Molecular Biology and Genetics, Democritus University of Thrace, University Campus-Dragana, 68100 Alexandroupolis, Greece; eltr.mailbox@gmail.com (E.T.); achlichl@mbg.duth.gr (K.C.); 3 Microbial Biotechnology-Molecular Bacteriology-Virology Laboratory, Department of Biochemistry and Biotechnology, University of Thessaly, 41500 Larissa, Greece; markoulatosp@gmail.com; 4 Department of Biochemistry, University of Cambridge, Sanger Building, 80 Tennis Court Road, Cambridge CB2 1GA, UK

**Keywords:** coronavirus, SARS-CoV-2, COVID-19, evolution, recombination, point mutations, pandemic, vaccines, spike

## Abstract

Coronaviruses (CoVs) constitute a large and diverse subfamily of positive-sense single-stranded RNA viruses. They are found in many mammals and birds and have great importance for the health of humans and farm animals. The current SARS-CoV-2 pandemic, as well as many previous epidemics in humans that were of zoonotic origin, highlights the importance of studying the evolution of the entire CoV subfamily in order to understand how novel strains emerge and which molecular processes affect their adaptation, transmissibility, host/tissue tropism, and patho non-homologous genicity. In this review, we focus on studies over the last two years that reveal the impact of point mutations, insertions/deletions, and intratypic/intertypic homologous and non-homologous recombination events on the evolution of CoVs. We discuss whether the next generations of CoV vaccines should be directed against other CoV proteins in addition to or instead of spike. Based on the observed patterns of molecular evolution for the entire subfamily, we discuss five scenarios for the future evolutionary path of SARS-CoV-2 and the COVID-19 pandemic. Finally, within this evolutionary context, we discuss the recently emerged Omicron (B.1.1.529) VoC.

## 1. Introduction

The outbreak of the coronavirus disease 2019 (COVID-19) pandemic, caused by the severe acute respiratory syndrome coronavirus-2 (SARS-CoV-2) [1,2,3], has raised several important questions. The most crucial one is how the currently circulating SARS-CoV-2 virus will evolve in the future: Will new mutations or recombination events, even with distantly related human or animal coronaviruses (CoVs) lead to new variants of concern (VoCs) or to variants of high consequence, or even to a new CoV species to which current vaccines may offer no protection? Another related question concerns the development of the next generation of CoV vaccines and how their long-term efficacies could be improved. In order to answer these and other questions concerning the origin of the virus and the possibility of the emergence of a new epidemic or pandemic in the future [4,5], it is of paramount importance to understand the evolution of CoVs as an entire group (subfamily). To that end, this review focuses on the unprecedented wealth of data generated over the last two years, not only for SARS-CoV-2 but for the entire *Orthocoronavirinae* subfamily.

## 2. Notable Coronaviruses

CoVs are found in a wide range of animal species, especially bats and birds. They cause from mild to severe respiratory, enteric, hepatic, or nervous system disorders [6,7]. So far, four of the human CoVs (HCoV-NL63, HCoV-229E, HCoV-OC43, and HCoV-HKU1) cause mild infections such as the seasonal common cold. In contrast, three other human CoVs (i.e., SARS-CoV-1 [8], Middle East respiratory syndrome coronavirus (MERS-CoV) [9], and SARS-CoV-2 [1,2,3]) have been implicated in severe respiratory and multi-organ disorders [7]. Other CoVs cause important diseases in farm animals and include swine acute diarrhea syndrome coronavirus (SADS-CoV), swine transmissible gastroenteritis virus (TGEV), porcine epidemic diarrhea virus (PEDV), and avian infectious bronchitis virus (IBV). The murine hepatitis virus (MHV) is one of the best characterized animal CoVs and is an important research model [7,10].

## 3. Classification

CoVs are enveloped, spherical, positive-sense single-stranded RNA viruses [11] that belong to the order *Nidovirales*, family *Coronaviridae*, and the subfamily *Orthocoronavirinae*. Members of the order *Nidovirales* include viruses with the largest RNA genomes, with sizes ranging from 13 to 41 kb [12,13]. Together with CoVs, toroviruses (the closest relatives of CoVs) and roniviruses constitute the “large” nidoviruses (with genomes sized >26 kb), whereas arteriviruses have much smaller genomes (13–16 kb) [12]. Nidoviruses display a substantial genomic plasticity and resilience [14].

Based on the 2019 International Committee on Taxonomy of Viruses (ICTV) report, the *Orthocoronavirinae* subfamily includes four genera: Alpha-CoVs with 14 subgenera and 19 species; Beta-CoVs with five subgenera (i.e., *Sarbecovirus, Hibecovirus, Nobecovirus, Merbecovirus,* and *Embecovirus*) and 14 species; Gamma-CoVs with three subgenera and five species; Delta-CoVs with three subgenera and seven species [15]. The taxonomy and species demarcations for CoVs were originally based on serological reactivities of the spike proteins, where CoVs were divided into three serogroups [16], but they are now based on genomic criteria and, more specifically, on five replicative peptides (i.e., 3CLpro, NiRAN, RdRp, ZBD, and HEL) common to all *Nidovirales*. Furthermore, their selection for species demarcation is based on observations that these regions are not involved in cross-species homologous recombination [3]. This taxonomic demarcation is achieved with the DEmARC software [17,18]. The cutoff for species demarcation is 90% amino-acid sequence identity in the abovementioned conserved replicase polypeptides.

In order to classify SARS-CoV-2 genomes, the different mutations that give rise to functional variants are further divided into higher order phylogenetic lineages and clades. Currently, three nomenclature systems are being used: the GISAID (Global Initiative on Sharing All Influenza Data), Nextstrain, and Pango [19,20,21,22]. As of 16 December 2021, more than 6.2 million SARS-CoV-2 genomes have been sequenced and deposited in the EpiCoV database of the GISAID [19]. An extensive review of the various clades, their characteristic mutations, and their phenotypic impact is provided in [23]. Variants are also classified by the World Health Organization (WHO) and the Centers for Disease Control and Prevention (CDC) into different categories based on their phenotype [24]. For example, VoCs, such as the Alpha, Beta, Gamma, and Delta SARS-CoV-2 variants (which should not be confused with the Alpha, Beta, Gamma, and Delta CoV genera), may have increased transmissibility, and/or increased pathogenicity, and/or reduced neutralization by antibodies, and/or reduced detection by diagnostic methods. However, the most notable case that has caused alarm around the world is the recently emerged Omicron VoC (B.1.1.529), which was first reported in November 2021. This variant has over 50 mutations compared to the original Wuhan strain, with over 30 of them located at the Spike ORF. The Omicron variant has already been observed to significantly outcompete the existing prevalent, and already very infectious, Delta variant. Omicron cases in the UK are doubling every 2 days, and this variant also has a very high reinfection rate (a 5.4-fold increase over that of Delta) [25]. Accordingly, 26 out of 29 existing monoclonal antibodies (mAbs) that target the receptor binding motif (RBM) cannot neutralize Omicron in vitro, although some mAbs that target antigenic sites outside of the RBM still can [26]. Whether Omicron causes lesser or more severe infections will soon be resolved.

The US Food and Drug Administration (FDA) closely monitors circulating variants that due to the specific mutations may escape detection by certain nucleic acid, antigen, or serology tests. For example, the Omicron variant already escapes detection by some standard tests [27]. In addition, certain nucleic acid tests that monitor two or three different regions of the virus may show an apparent absence of target sequences within Spike or nucleocapsid genes. While such failures raise suspicions that the sample contains the Omicron variant, other variants may also cause this pattern of failure in gene detection [27].

## 4. Distribution

CoVs have become a focal point of virus research, especially after the 2002–2003 SARS-CoV-1 epidemic. Data collected over the last two decades show that bats and rodents serve as reservoirs for the Alpha and Beta CoV genera, whereas wild birds probably serve as reservoirs for the Gamma and Delta CoV genera [28,29,30,31]. The human CoVs have a zoonotic origin, where bats and rodents seem to be the key reservoirs [32,33] with intermediate hosts, such as civets, raccoon dogs, camels, and possibly (the evidence here is weak) pangolins, playing a role in cross-species transmission [31,34,35,36]. The recent detection of porcine delta coronavirus (PDCoV) and a feline–canine recombinant Alpha-genus CoV in human patients demonstrate that many more animals may actually serve as either reservoirs or intermediate hosts [37,38]. Other cases of cross-species transmission include the emergence of HCoV-OC43 (most probably from a bovine or swine CoV), whereas the Embecovirus HCoV-HKU1 is a sister taxon to MHV and rat sialodacryoadenitis virus [32,39].

The animal receptors to which CoVs bind, such as angiotensin-converting enzyme 2 (ACE2) and aminopeptidase N (APN), are highly conserved in evolutionary terms, and this leads to a high incidence of cross-species infection including from birds to mammals. Such transmission events may either be direct or happen via a number of hosts at intermediate points on the evolutionary scale [31,40,41,42,43,44,45,46]. Spill-back infections from humans to other animals (domesticated or wild) that are termed reverse zoonoses have been reported and should be monitored thoroughly [47]. For example, SARS-CoV-2 is able to infect gorillas, dogs, cats, lions, tigers, pumas, cougars, snow leopards, ferrets, deer, and minks [31,48]. There have been cases of SARS-CoV-2 transmission from humans to minks and back to humans [49]. Such events are matters of concern, since mutations acquired in minks might lead to the emergence of vaccine-escaping variants [50]. Concerning the COVID-19 pandemic, the animal reservoir for SARS-CoV-2 is considered (so far) to be the *Rhinolophus* (horseshoe bat) spp., residing in the wider area of Indochina and Southwest China [46]. However, the documented reverse zoonosis and sustained infection events, especially in deer and minks, suggest that new animal reservoirs may also be established [48,49]. The events that have led to the emergence of the current pandemic virus are still under investigation [4,5].

## 5. Genome Architecture

CoVs have very large genomes compared to other RNA viruses, with a length of 25–32 Kb, consisting of a 5′ untranslated region (UTR), at least six core open reading frames (ORFs) found in all CoVs, a highly variable number of accessory ORFs, and a 3′ UTR [7,12,51,52]. This large genome size may provide scope for the evolutionary flexibility required for cross-species adaptation [53]. The genome architecture of CoVs follows a model of division of labor [53].

The first two ORFs, 1a and 1b (which overlap by a few nucleotides), span two-thirds of the genome and encode polyproteins that are cleaved into 16 non-structural peptides (NSPs 1–16), most of which are involved in transcription and replication. ORF1a (NSPs 1–11) is considered to orchestrate the expression of the entire genome, and it also encodes two proteinases. ORF1b (NSPs 12–16) encodes the principal enzymes involved in RNA synthesis; these include the RdRp (NSP 12), a helicase (NSP 13), an exonuclease (NSP 14), an endoribonuclease (NSP 15), and a methyl-transferase (NSP 16) [53,54].

The downstream ORFs control genome dissemination [53]. More specifically, the Spike ORF (S) encodes a structural protein that binds to the host receptors and determines the types of cells that can be infected (cell tropism) [55,56,57,58] as well as the host range [54,59]. Next, the envelope ORF (E) encodes a protein that is involved in viral envelope curvature, maturation, assembly, release, and pathogenesis [54]. It is followed by the M ORF that encodes a membrane protein that interacts with the virion proteins S, E, N, and the viral genomic RNA, nucleating these components at the budding endoplasmic reticulum–Golgi intermediate compartment [54]. The nucleocapsid protein is an RNA-binding protein, encoded by the subsequent ORF N [54]. Thus, the latter four (i.e., S, E, M, and N) of the abovementioned core ORFs each have a structural role.

Lineage-specific accessory ORFs are also present in this virus-dissemination region of the genome, and they may be involved in adaptation to specific hosts, modulation of the interferon signaling pathways, the production of pro-inflammatory cytokines, or the induction of apoptosis [12,51,54,60,61]. The accessory ORF6 displays the highest cellular toxicity among all core and accessory ORFs of SARS-CoV-2 [62]. Two recent large-scale computational analyses shed light on the distinct genome architectures and the dynamic evolution of accessory ORFs [52,63].

## 6. Evolution by Point Mutations

CoVs are unusual RNA viruses in possessing a replication proof-reading mechanism conferred by the NSP 14 exonuclease, an enzyme found only in “large” *Nidovirales*. This significantly reduces their mutation rate to a level that is similar to that of DNA viruses and, thus, allows them to develop some of the largest genomes among all other RNA viruses [42,53,64,65,66,67]. A recent experimental evolution study under relatively benign conditions estimated a background mutation rate of 2.9–3.7 × 10^−6^/nt/replication cycle for SARS-CoV-2 [68]. This rate is similar to that of another Beta-genus CoV, mouse hepatitis virus (MHV: 3.5 × 10^−6^/nt/replication cycle) [69]. Interestingly, the Spike gene has a mutation rate at least 4–5 times higher than the rest of the genome [68]. The large number of mutations observed in the spike (34/53 total mutations) of the recently emerged Omicron VoC is congruent with the above studies. At the same time, the relatively low mutation rate of CoVs is also considered their “Achilles’ heel” and has been exploited in order to produce the first approved (by the UK) SARS-CoV-2 orally administered antiviral drug: a ribonucleoside analog that introduces copying errors and, thus, dramatically increases the mutation rate of many diverse CoVs (including MERS-CoV) to a lethal level beyond the error threshold [66,67,70,71,72].

CoVs would not be expected to rapidly adapt to new environments and hosts via point mutations, unless they have some mechanism that allows them to regulate and increase their mutability in the first phases of host transition. However, the effects of even a few point mutations should not be underestimated, since they can be sufficient to transform a CoV strain with mild symptoms into a strain that may change cell tropism and induce severe systemic pathology. Point mutations (especially in the gene for the spike protein) are capable of increasing replication, transmissibility, and even lead to immune escape [73,74,75]. A notable example is a Feline CoV that mutates to a lethal form named feline infectious peritonitis virus (FIPV) by a few point mutations in the C-terminal part of the Spike gene [58]. These mutations affect cell entry and cause a change in cell tropism, from enteric epithelia to macrophages [58].

Thanks to new-generation sequencing technology, an unprecedented number of SARS-CoV-2 genomic sequences have garnered. This plethora of data has allowed, for the first time, in-depth microevolutionary analyses and monitoring of the emergence, spread, and adaptations of new lineages/strains during the course of an epidemic or pandemic [76,77,78,79,80]. Molecular clock analyses suggest that SARS-CoV-2, within the first year of the pandemic, displayed a slightly higher mutation rate than SARS-CoV-1, MERS-CoV, or HCoV-OC43. This is in agreement with a phenomenon termed time-dependent rate variation, where slightly deleterious mutations tend to be purged in the later stages of an epidemic [76,81]. However, SARS-CoV-2 did not appear to have undergone significant mutational changes within the first year of its spread in the human population [82], and this poses difficulties for phylogenetic analyses [83].

A very interesting study analyzed 192,000 SARS-CoV-2 genomes that were sequenced in the first year of the pandemic (as of December 2020) and focused on non-synonymous mutations, their distribution across the genome, and their functional/structural effects [84]. The study observed regions of low and high non-synonymous mutations, even within the same gene. Overall, ORF1ab was subject to lower mutation rates compared to the structural and accessory ORFs. The genome regions encoding for the nucleocapsid-structured regions/domains also had significantly reduced mutation rates. Reassuringly, these observed microevolutionary patterns of mutation rates for the various genomic regions of SARS-CoV-2 are in agreement with divergence at the macroevolutionary/genus level [52]. Furthermore, the authors of that study argue that the residues of the spike receptor-binding domain (RBD) that come into contact with ACE2 are under strong purifying selection [84]. Thus, the epitopes for these residues are “protected” from mutations, meaning that antibodies targeting such conserved epitopes are at a lower risk of immunological escape by the virus [84]. In conclusion, structural constraints (i.e., tightly packed cores and protein–protein interaction surfaces) and genome organization (i.e., regions of overlapping ORFs) are drivers of purifying selection in SARS-CoV-2 [84]. Indeed, the programmed frameshift element and its related RNA secondary structure elements, situated at the overlap between ORF1a and ORF1b, are highly conserved [85].

Interestingly, another study observed the occurrence of recurrent missense mutations in certain regions of SARS-CoV-2, a sign of adaptation and convergent evolution [86]. An experimental evolution study of SARS-CoV-2 also confirmed the occurrence of several convergent mutations, especially at the Spike ORF [68].

## 7. Evolution by Insertions/Deletions

Apart from point mutations, insertions and deletions (indels) also occur frequently. Large-scale SARS-CoV-2 sequencing data (>1.7 million genomes) coupled with sophisticated bioinformatics analyses have revealed many such events, which are located more frequently at the 3′ half of the genome. These occur especially at the spike region and have the potential to lead to escape from neutralizing antibodies or even T-cell immunity [87]. For example, the Omicron variant has three deletions and one insertion within the spike and three deletions in other genomic regions. A non-coding deletion in the B.1.1.7 SARS-CoV-2 lineage increases the translational efficiency of the accessory ORF9b (an interferon antagonist); it interacts with other non-synonymous point mutations and increases viral transmissibility [88]. Spike-located inserts have also been associated with increased pathogenicity [89]. A notable example is the polybasic 4-amino-acid insertion of an initially suboptimal furin cleavage site (FCS) found in SARS-CoV-2 that plays a significant role in its transmission. This FCS has not yet been found in other sarbecoviruses, but it is present in other Beta-CoV subgenera [4,5,90,91]. One hypothesis about the origin of the FCS is that it was generated naturally by a template switch, followed by substitutions that eventually erased its similarity to the original sequence [87]. The FCS sequences of several CoVs resemble polybasic low-complexity regions that tend to evolve fast [92]. Notably, the Omicron variant bears two mutations (i.e., N679K and P681H) at the FCS. Alternative theories about the natural emergence of the FCS in SARS-CoV-2, perhaps involving recombination events with as yet unknown FCS-bearing sarbecoviruses or even with other Beta-CoV subgenera that cannot be excluded [52].

## 8. The Progenitor of SARS-CoV-2 Was Already a Generalist Virus That Did Not Need Many Mutations to Adapt to Its Human Hosts

Evolutionary and experimental studies show that the bat SARS-CoV-2 progenitor had already evolved into a generalist virus that had the ability to efficiently spread to other mammals. This is probably the reason that the virus did not undergo very strong positive selection during the first year of the pandemic [82]. Interestingly, a study that used surrogate entry assays and live virus showed that the SARS-CoV-2 spike protein has a broad host tropism for many mammalian ACE2 receptors (especially for those of cattle, cats, and dogs), whereas its tropism is significantly reduced for bat and bird ACE2 receptors [93]. Therefore, an intermediate host/reservoir is likely to have been required to allow sufficient point mutations and or insertions/deletions to occur in order to shift receptor usage from bats to humans. On the contrary, another very recent study identified the closest (so far) relative of SARS-CoV-2 in *Rhinolophus* bats from Laos [46]. The RBD of this *Rhinolophus* spike is very similar to the SARS-CoV-2 spike and is capable of binding to the human ACE2 receptor with an affinity that is similar to that of the original Wuhan strain. However, the isolate from Laotian bats lacks the FCS. Other *Sarbecovirus* spike proteins from bats have also demonstrated a capacity to bind to the human ACE2 receptor, and their carrier CoVs have been shown to be able to infect a wide range of cell lines including human airway cells [44,94]. A recent in vitro study revealed that the Omicron spike has, for the first time, acquired binding to mouse ACE2 [26], confirming the remarkable evolutionary plasticity of this region.

## 9. Evolution by Recombination

Although CoVs have a relatively low mutation rate compared to other RNA viruses, they also display a relatively high recombination rate [95]. The NSP s14 exonuclease is responsible for proof-reading and, thereby, the fidelity of genome replication. However, this enzyme also mediates recombination, whereas its inactivation decreases the frequency and also alters the patterns of recombination [65]. CoVs, like many other RNA viruses, tend to recombine frequently, using a template switching mechanism [96,97,98,99]. In addition, CoV transcription involves sub-genomic mRNAs that are formed by template switching among the transcriptional regulatory sequences (TRS) of the various ORFs (designated TRS-B: B for body) and the TRS at the 5′UTR (designated TRS-L: L for leader) [100,101]. Therefore, CoVs are inherently prone to recombination due to the fact of this characteristic transcription mechanism [102].

Many studies have highlighted the crucial role of homologous recombination in the evolution of CoVs, and these were extensively reviewed before the COVID-19 pandemic [97,103]. However, the plethora of new data and evolutionary/genomic analyses published in the last two years has significantly increased our understanding of the recombination events between both closely and distantly related CoVs. These recombination events may occur among members of the same strain/species, among members of the same subgenus, or even among members of different subgenera of the same genus. Furthermore, non-homologous recombination may occur even among CoVs and other viruses or even hosts. Recombination events, especially in certain regions such as spike, may result in changes in cell tropism and host range [44,59], with particular examples concerning MHV [55], TGEV [56], IBV [57], and FCoV/CCoV [38,58].

## 10. Evolution by Intratypic Homologous Recombination

Recombination among closely related strains/genotypes and species of the same subgenus occurs readily due to the high sequence identity throughout the genome and may result in the emergence of new strains [76,95,98,103,104,105,106,107,108,109,110,111]. We use the term intratypic to describe this category of recombination. Early analyses determined that in mammalian cells, recombination frequently occurs among regions that share identical stretches of ~200 base pairs and, furthermore, that recombination could occur at very low frequency between regions of sequence identity as small as 14 base pairs [112]. More specifically, CoVs have an intrinsically high intratypic recombination rate of approximately 25% across the genome. The crossover sites may occur anywhere, but selection pressure can lead them to cluster in certain hotspots [99,113].

Several recent computational studies have analyzed available genomes from most CoV subgenera in order to better understand the patterns of intratypic recombination [76,95,107,108,111,114,115]. In one recent study [108], phylogenetic analyses of the various genomic regions revealed many incongruities among genomes of the same subgenus. These data were further analyzed with recombination detection program 4 (RDP4) and revealed 973 intratypic recombination events within 16 different subgenera [108]. Although these intratypic crossovers were scattered throughout the genome, and many of them were localized in hotspots that were, in turn, situated in the neighborhood of TRSs, especially preceding the Spike ORF. This region has long been considered as a modular recombination hotspot [42,52].

Evolutionary analyses of Sarbecovirus genomes, using the RDP5 and GARD (genetic algorithm for recombination detection) programs, identified many intratypic recombination events with a hotspot upstream of the Spike ORF and two cold spots in S1 and ORF8 [111]. This pattern may be attributed to antigenic selection in ancestral viruses, since the immunodominant N-terminal domain and receptor-binding motif regions are within the S1 subunit. Another recent analysis focused on sarbecoviruses, embecoviruses, and SADS-CoV lineages and identified many recombination events within each lineage where the donor was unknown [95]. This is a clear indication that the sequence analysis of currently available samples has underestimated CoV diversity. In addition, that study also revealed that many of the intratypic recombination events occur within and around the Spike ORF.

One challenge when analyzing very similar genomes is how to distinguish recombination from undetected fast evolution, accompanied by positive selection, that leads to convergent mutations. Recombination events in three of the five Beta-CoV subgenera (e.g., sarbecoviruses, merbecoviruses, or embecoviruses) have been examined, with a particular focus on the h/m ratio (homoplastic/non-homoplastic polymorphisms) [107]. This investigation determined that intratypic recombination events tend to localize around the spike region and account for 40% of the observed polymorphisms. Furthermore, the highest rates of intratypic recombination were found within embecoviruses in contrast to a similar study that found sarbecoviruses recombine with much higher frequency than any of the other two subgenera [115].

A survey (using RDP4) of ~158,000 human CoV genomes (SARS-CoV-1, SARS-CoV-2, MERS-CoV, HCoV-OC43, HCoV-HKU1, HCoV-NL63, and HCoV-229E) that looked for signs of intratypic recombination [114] identified several high-confidence events in various human CoVs. Only eight recombination events of moderate confidence were identified among ~157,000 SARS-CoV-2 genomes. The majority of these events were located in the structural genes. It was also estimated that the time of emergence of each of the currently circulating human CoVs was within the last 70 years and, especially, for HCoV-229E, continuous lineage replacements were observed.

Recombination events among currently circulating SARS-CoV-2 genomes should also occur. However, it would be expected [116] that such events would be difficult to detect at the start of the pandemic due to the high level of sequence identity among the circulating genomes. But as the pandemic progressed and more divergent SARS-CoV-2 strains were circulating, it should become more possible to detect intra-SARS-CoV-2 recombination events [117]. Indeed, several studies have already reported such events [114,117,118,119,120]. An analysis of 1.6 million SARS-CoV-2 genomes showed that 2.7% of circulating sequences belong to a recombinant lineage and that the recombination breakpoints occur disproportionately at the spike region [119]. As more point mutations occur in the various SARS-CoV-2 variants, there is the possibility of an even greater increase in the diversity of this species via intratypic recombinational shuffling. Therefore, SARS-CoV-2 may still be within the first phase of a rather slow evolution that is mostly driven by point mutations and insertions/deletions. This may be followed by another phase of rapid divergence driven by a combination of point mutations/insertions/deletions and recombinational shuffling among the different lineages with significant implications for vaccine efficacies.

A question of great importance concerning the current SARS-CoV-2 pandemic is how it emerged, and whether a recombination event involving a bat Sarbecovirus resulted in its evolved ability to efficiently infect humans and other mammals. Studies conducted during the first phase of the pandemic suggested that SARS-CoV-2 is the product of recombination, where a close relative of a bat SARS-related genome (RaTG13) acquired the receptor-binding motif of the Spike gene of a pangolin SARS-related virus [36,121]. However, subsequent analyses suggested that no recent recombination event led to the emergence of SARS-CoV-2 [76,122]. Instead, its bat ancestor already had evolved the necessary amino acids in the RBD that allowed it to act as a generalist and infect many other mammalian cells via the ACE2 receptor [76]. Similarly, another study [123] applied ancestral sequence reconstruction and structural modeling and concluded that an old recombination event occurred several decades ago at a common ancestor of SARS-CoV-1 and SARS-CoV-2 that led to the emergence of an RBD with an increased binding affinity for human ACE2. Thus, that ancestral Sarbecovirus and its descendants already had the ability to infect humans. All the above conclusions are further supported by the detection, in what is the closest progenitor to SARS-CoV-2 so far recognized, of *Rhinolophus* sp. found in Laos. Intriguingly, its spike is capable of binding with high affinity to human ACE2, but it lacks the FCS [46].

Recombination between members of the SARS-CoV-1 and SARS-CoV-2 lineages within the sarbecoviruses has also been observed [52,76,111]. This is a matter of great concern, since it demonstrates a potential for the future emergence of a SARS-CoV-3 that may combine the high pathogenicity of SARS-CoV-1 with the high infectivity of SARS-CoV-2.

## 11. Evolution by Intertypic Homologous Recombination

Homologous recombination among more distantly related CoVs from different subgenera is expected to occur infrequently, since their genomes share less than 50% nucleotide identity [108]. In addition, due to the fact of this low sequence identity and the undersampled CoV diversity, it will be difficult for many recombination software packages to detect old recombination events among different subgenera or even recent events involving as yet unsequenced subgenera, because the closest available relative of the unsequenced donor will still be quite divergent. Phylogenetic incongruence is a popular and robust method for such types of analyses [124]. Nevertheless, recombination among different CoV subgenera does happen, and we call such events intertypic. The most recent and striking example involves the emergence of swine enteric coronavirus due to the cassette-like recombination of the spike region between PEDV and TGEV, which belong to two different subgenera (i.e., *Pedacovirus* and *Tegacovirus*) of Alpha-genus CoVs [125].

Intertypic recombination between CoVs is possible due to the presence of the highly conserved transcriptional regulatory sequences (TRS) at the beginning of the various ORFs [100,101,102,125,126]. The main role of these TRSs is to facilitate template switching during transcription to generate sub-genomic (sg) RNAs [100,101]. In addition, these highly conserved TRSs could potentially function as recombination hotspots among distantly related strains/species [102]. At the same time, they may also function as barriers to recombination between lineages that possess very different TRSs [127]. Based on these hypotheses, there have been studies to engineer live attenuated CoVs with altered TRSs that will not be able to recombine with other CoVs and, thus, block reversion and vaccine-derived epidemics [102]. Cases of live attenuated vaccine strains that reverted to pathogenic ones via recombination with wild-type close relatives have been observed for poliovirus vaccines [128,129,130,131,132,133].

The most extensive large-scale analysis of intertypic recombination undertaken so far included 196 representative genomes from all fully sequenced CoV subgenera [52]. This analysis was based on the phylogenetic incongruence of 15 NSPs of ORF1ab (NSP 11 was excluded due to the fact of its short length) and the other four core ORFs (i.e., spike, envelope, membrane, and nucleocapsid). The analysis identified several phylogenetic incongruities supported by neighbor joining, PhyML, Bayesian trees, and by the Shimodaira–Hasegawa test. The observed incongruities were consistent with old as well as recent recombination events that occurred at the origin of certain subgenera or even later. The vast majority of these events were detected at the spike region, highlighting it as a recombination hotspot even among different subgenera. These spike-localized recombination events were observed in five Alpha-CoV subgenera, one Gamma-CoV subgenus, and all three Delta-CoV subgenera. Furthermore, several donors of intertypic events in Gamma and Delta genus CoVs most probably involve subgenera that have yet to be sequenced. This is in agreement with another recent observation that the currently known CoV diversity is only an underestimate and that many more lineages wait to be discovered [95].

Intriguingly, no well-supported intertypic recombination event of the spike region was observed among any of the five Beta-CoV subgenera [52], but there was an incongruence concerning the nucleocapsid region of merbecoviruses. The same lack of intertypic recombination among Beta-CoV subgenera was also reported in [107,108]. This observation is of great importance because, so far, it does not appear that SARS-CoV-2 is able to recombine with any of the other circulating human CoVs that belong to different subgenera and genera. MERS-CoV is a Beta-CoV Merbecovirus, HCoV-HKU1 and HCoV-OC43 are Beta-CoV embecoviruses, and HCoV-NL63 and HCoV-229E are Alpha-genus CoVs. However, a recent theoretical analysis based on syntenic homologous regions has shown that there exist potential recombination crossover sites approximately 30–40 nucleotides long with very high sequence identity between the SARS-CoV-2 genome and that of its closest circulating human CoV, MERS-CoV; these are situated in ORF1b and the spike S2 region [126]. It may be proposed that intertypic recombination events between these two viruses could occur in the gastrointestinal tract, where both receptors (i.e., ACE2 for SARS-CoV-2 and dipeptidyl peptidase-4 (DPP4) for MERS-CoV) are highly co-expressed. In such an evolutionary scenario, the intertypic recombinant could possibly change its tissue tropism and/or pathogenicity by undergoing further adaptive evolution. This might render the ongoing vaccination programs obsolete due to the fact of their focus on certain genomic regions and, especially, the spike ORF, which tends to evolve rapidly (both by point mutations and recombination). These observations [126], together with the evidence presented in [52] for the high intertypic recombination potential in other CoV genera, are a significant cause for concern and vigilance.

## 12. Intertypic Recombination Is Modular

Another important observation in [52] was that the intertypic recombination events did not occur as single crossovers but instead occurred as double crossovers, meaning that a sequence module or cassette was exchanged. Our interpretation is that several genomic features prevent (or select against) the occurrence of such intertypic single crossover events: (i) the long-range genetic/physical interactions within certain elements of a CoV genome and (ii) the distinct accessory ORF architectures of the various subgenera (especially in Beta-genus CoVs).

Reverse genetic experiments have shown that the N protein, encoded by an ORF at the 3′ of the genome, is essential for replication and have also demonstrated that, while N proteins from different members of the same genus may be at least partially compatible, those from different genera are functionally incompatible [134,135]. One such example is PEDV-N against TGEV-N and PDCoV-N proteins [135]. In addition, N proteins from other genera may even have a suppressive effect, which has serious implications for CoV co-infection from different genera [135]. The N protein may also be involved in circularization of the genome by acting as a bridge between the 5′ and 3′ ends [136]. Furthermore, RNA secondary structures have been shown to form long-range interactions within the genome [137] and to interact not only with viral but also cellular components in order to initiate transcription and replication [138]. Genetic interactions have been observed between the non-structural peptides NSP 8, NSP 9 (encoded at the end of ORF1a), and the pseudoknot at the 3′ end of the genome [139]. Recently, a protein interaction map was generated for SARS-CoV-1, SARS-CoV-2, and MERS-CoV peptides based on affinity purification/mass spectrometry in HEK-293T/17 cells, and it revealed 366, 389, and 296 protein interactions with human proteins [140]. Only 21% of the interactions were shared by all three viruses, 45% of interactions were unique for each virus, whereas SARS-CoV-2 and MERS-CoV shared only 24% of interactions. Accordingly, a single crossover intertypic recombinant between SARS-CoV-2 and MERS-CoV would most probably be inviable, partly due to the protein–protein interaction incompatibilities. Thus, single crossover recombination events among different subgenera may break/disrupt such genetic or even physical interactions, whereas double-crossover/cassette-like events are likely to retain compatible interactions.

## 13. Evolution by Non-Homologous Recombination among CoVs and with Other Taxa

Apart from homologous intratypic and intertypic recombination events, non-homologous recombinations also occur in CoVs, leading to the acquisition of accessory genomic regions by gene duplication or from other CoVs, viruses, and even hosts [52,63]. A recent evolutionary study developed PSI-Blast profiles for 73 non-redundant CoV accessory ORF families (AOFs), permitting the performance of a very sensitive homology search [52]. The study observed that many of these AOF families had homologs found only in certain CoV subgenera or genera. Each genus and, in certain cases, some subgenera (especially in the Beta-genus CoVs) had very distinct accessory ORF architectures [52,63,141]. Such a pattern could be explained by either the “birth” of genes de novo in the common ancestor of certain genera/subgenera, the rapid divergence of existing ORFs and loss of the homology signal, or via non-homologous recombination with ORFs (followed by rapid divergence) from other CoVs, viruses, or even hosts [52,142,143,144,145].

Evolutionary studies have identified several non-homologous recombination events in CoVs involving gene duplications. Such events include a bat Beta-CoV *Hibecovirus* ORF2 and a *Luchacovirus* ORF6, originating from spike [52]. Another gene duplication event concerns the ORF3a of Beta-CoV *Sarbecovirus/Hibecovirus/Nobecovirus* that appears to have originated from the core membrane ORF (followed by rapid divergence) [52,144] or SARS-CoV-2 ORF8 originating from ORF7a [145].

The study presented in [52] found evidence (and also confirmed previous observations) of horizontal gene transfer for seven AOFs that had homologs in other taxa, outside of the CoVs; three of them were localized in the vicinity of spike. The best studied, striking, and rather worrying example concerns a hemagglutinin-esterase found in Beta-CoV *embecoviruses*, situated just before the spike region. This gene was transferred from an influenza C/D virus, either directly or via a Torovirus that then adapted and co-evolved with the spike [146,147,148,149]. Other examples include genes for a phosphodiesterase (probably originating from toroviruses) [52], a C-type lectin (probably originating from mammals) [150], a p10-like gene (probably originating from reoviruses) [151], an NSP-1-like gene (probably originating from avian rotavirus-g), a uridine kinase (originating from a whale host) [152], and a distant homolog to the capsid protein of human astrovirus 5. Therefore, toroviruses emerge as a key taxon, not only because they belong to the same order (i.e., *Nidovirales*) as CoVs, but also because they can act as gene donors in other viral orders as well, e.g., porcine *enterovirus-*G [153,154]. Given the worldwide distribution and high infection rate of toroviruses [153], many more non-homologous recombination events between them and their close relatives, the CoVs, may be detected in the near or more distant future.

## 14. Implications for Vaccine Design and Development

As of Autumn 2021, more than twenty COVID-19 vaccines have received emergency use authorization in at least one country, with efficacies in the range of 66–95% [155]. These vaccines are grouped into two major categories: the ones based on the inactivated virus and the ones that are based on the spike DNA/mRNA or spike protein [156,157]. Vaccines in former category should train the immune system with many diverse SARS-CoV-2 epitopes; however, virus aggregate formation or protein denaturation/degradation or crosslinking during the inactivation process may significantly reduce their efficacy [158]. So far, vaccines in the latter category demonstrate higher efficacies [155]. At the same time, the spike-focused vaccines are vulnerable to mutations in a region of known genetic instability. Such mutations have previously led to the emergence of VoCs that are already capable of partly evading the immunity conferred by current vaccines and, over time, may render these vaccines ineffective [159,160,161,162,163]. Worryingly, the recent emergence of the Omicron (B.1.1.529) VoC, which has an unusually high number of mutations located in the spike region, raises great concern regarding the efficacies of current vaccines against this VoC. Epidemiological surveillance data from South Africa and the UK already show an increased risk of reinfection by Omicron [25,164]. Most mAbs that target antigenic sites at the highly mutated RBM are already unable to neutralize the Omicron VoC in vitro [26]. The problem may soon be exacerbated by intratypic or even intertypic recombination events in the spike region that could lead to further diversity [52,95,107,108,111]. On the other hand, the fast development (within a few months) of new vaccines based on current mRNA vaccine platforms allows for an unprecedented rapid and effective response to new variants.

To address this looming problem, the National Institute of Allergy and Infectious Diseases (NIAID) in the USA has recently awarded three projects for the development of pan-coronavirus vaccines that will provide broad protective immunity to multiple CoVs. All three projects focus on the spike protein but specifically on conserved regions that may cover all sarbecoviruses or even all Beta-genus CoVs. Furthermore, such vaccines would be an insurance against any future intertypic recombination events where a highly contagious SARS-CoV-2 variant acts as a spike donor to a human or animal CoV of the same or different subgenus, thus transforming it into a highly contagious human pathogen. The spike vaccine approach that is targeting more CoV taxa is promising as several studies have already shown [165,166,167,168]. For example, pan-Sarbecovirus neutralizing antibodies have been observed in people that were originally infected by SARS-CoV-1 in the 2002–2003 epidemic and were later immunized with the BNT162b2 mRNA vaccine that targets the SARS-CoV-2 spike [167]. In addition, an immunodominant region located at the C-terminal fusion peptide domain of the SARS-CoV-2 spike can be recognized by CD4^+^ T cells that are generated by other common cold human CoVs [168]. Interestingly, some mAbs that target antigenic sites outside the RBM are capable of neutralizing even the highly mutated Omicron VoC in vitro [26].

The spike protein, especially its RBD, is a favored vaccine target, because it is capable of generating both a strong B-cell and T-cell response [169,170]. However, other protein (e.g., the nucleocapsid) are also capable of generating a robust T-cell response [171]. Moreover, the nucleocapsid gene displays rapid and high expression, high sequence conservation, and a low propensity for recombination [52,84,172]. On the other hand, early work on SARS-CoV-1 demonstrated that vaccines based on the nucleocapsid were ineffective and also enhanced the immunopathology in the lungs of senescent mice upon viral challenge [169]. Nevertheless, the SARS-CoV-2 nucleocapsid is already being tested as a vaccine target on its own, or in combination with the spike, or even with a third ORF as well [173,174,175,176,177,178]. Three such candidate vaccines (one in the USA and two in the European Union) have already reached Phase 2 clinical trials as of November 2021 [156].

A study of healthcare workers has also shown that pre-existing T cells against the early-transcribed replication transcription complex (RTC) of other human CoVs rapidly expand upon SARS-CoV-2 exposure, leading to an abortive infection [179]. Thus, vaccines against the highly conserved RTC may be capable of covering a broad spectrum of CoVs. Genes encoding components of the RTC are highly conserved and have a low propensity for recombination among the various CoVs [52]. In our opinion, future effective vaccines will target more than one region that contains conserved epitopes. The unexpectedly high number of mutations at the spike of the Omicron VoC also supports the strategy of investigating regions other than spike as vaccine targets.

## 15. Implications for Drug Design and Development

Projects to develop new drugs or repurpose existing ones have identified many candidates for the treatment of COVID-19 [180]. As of November 2021, two drugs have obtained government approval—Remdesivir and Molnupiravir [181]—whereas, a third candidate drug (i.e., Paxlovid) is also expected to obtain approval soon. Remdesivir is a ribonucleotide analogue that inhibits the RdRp and causes termination of viral replication [182]. Molnupiravir is a ribonucleoside analogue that introduces copying errors and, thus, dramatically increases the mutation rate of many diverse CoVs [71,72]. Paxlovid is a SARS-CoV-2 specific 3CL protease inhibitor that binds at the enzyme’s catalytic site. Mutations could arise that render any of these drugs ineffective, as has happened for human immunodeficiency viruses (HIV). For example, protease inhibitor monotherapy with ritonavir in HIV-positive patients led to the emergence of drug-resistant mutations (of the viral protease) that appeared in an ordered, stepwise fashion [183]. However, unlike HIV, SARS-CoV-2 infections are not chronic, and given the relatively low mutation rate, the drug-resistant mutants would not easily emerge within the patient. In addition, any drug-resistant mutants would also need to retain a fitness that is similar to that of wild-type strains in order not to be outcompeted. Remdesivir-resistant mutants have been circulating with a very low frequency in the general population; in vitro, they have emerged after several serial passages and display reduced fitness [182]. For all that, we would suggest that it might be wise to exclude viral proteins that are key drug targets from the list of targets for new vaccines. This is because antiviral drugs, in contrast to vaccines, will only be used on a minority of patients, and the inclusion of their target proteins in vaccine preparations will introduce considerable selection for the mutation of the viral genes that encode them.

## 16. Five Scenarios for the Future Evolution of SARS-CoV-2 during the COVID-19 Pandemic

**Figure 1:**
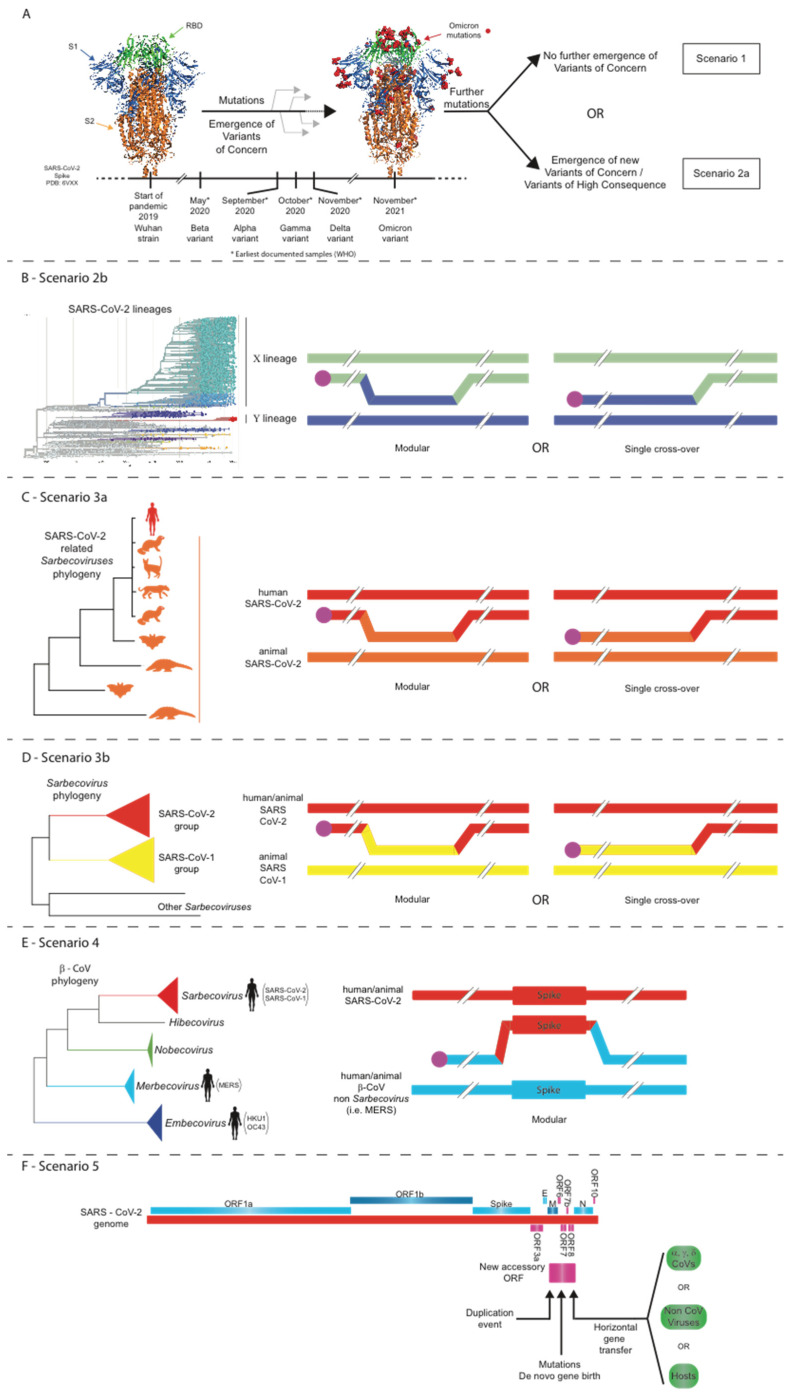
Five scenarios for the future evolutionary trajectory of SARS-CoV-2. (**A**) Scenario 1: structural constraints limit any further evolution of the SARS-CoV-2 spike; Scenario 2a: point mutations, insertions/deletions, and/or intra-SARS-CoV-2 recombination events lead to the evolution of novel SARS-CoV-2 strains. (**B**) Scenario 2b: intra-SARS-CoV-2 recombination events lead to the evolution of novel SARS-CoV-2 strains. (**C**) Scenario 3a: intratypic recombinations between SARS-CoV-2 and closely related sarbecoviruses. (**D**) Scenario 3b: intratypic recombinations between SARS-CoV-2 and other related sarbecoviruses. (**E**) Scenario 4: intertypic recombination between SARS-CoV-2 and viruses from other Beta-CoV subgenera. (**F**) Scenario 5: non-homologous recombination of SARS-CoV-2 with other coronaviruses or even other viruses/hosts.

Based on the observed data and evolutionary analyses for all CoVs so far, and especially the wealth of data generated in the last two years for SARS-CoV-2, we envisage several scenarios (some of high concern) for the evolution of SARS-CoV-2 during the current COVID-19 pandemic (see Figure 1). These scenarios could also be applicable to any future epidemic involving a novel CoV.

### 16.1. Scenario 1: Structural Constraints Limit Any Further Evolution of the SARS-CoV-2 Spike

In this scenario (Figure 1A), structural constraints [84] should not permit any further major mutations in the spike region that make the virus more infectious or significantly change the epitopes that are targeted by the current vaccines. Accordingly, as more people acquire immunity either via natural infection or spike-targeted vaccination, the COVID-19 pandemic will be controlled. The observed waning neutralizing antibody response generated by current vaccination schemes will make necessary a third or even regular booster doses, but some level of protection should continue to exist in vaccinated and previously infected people. In the longer term, immunization and/or protection by natural infection together with available drugs would lead to the transition of the pandemic into a seasonal (low-mortality) epidemic, similar to influenza. A protective effect against SARS-CoV-2 has been observed by cross-reactive CD4^+^ T cells that recognize the C-terminal section of spike and were elicited by other common cold CoVs [168]. The effective deployment of antiviral drugs, such as Molnupiravir, which recently gained approval in the UK, is also likely to promote the transition of the COVID-19 pandemic into a seasonal epidemic. The impact of long-COVID syndrome should also be seriously considered [184,185]. However, the recent emergence of the highly infectious Omicron VoC, with more than 30 new mutations in the spike ORF, demonstrates that the evolutionary flexibility of this region is significantly greater than was originally believed. Therefore, Scenario 1 may not be as feasible as it appeared before the emergence of Omicron. If Omicron or any future variant is more transmissible and significantly less pathogenic, then natural infections together with available drugs might lead to herd immunity and to the end of the pandemic.

### 16.2. Scenario 2: Point Mutations, Insertions/Deletions, and/or Intra-SARS-CoV-2 Recombination Events Lead to the Evolution of Novel SARS-CoV-2 Strains

In this scenario, many more people continue to become infected, especially in the developing world, where vaccination rates remain very low. Thus, the emergence of a new SARS-CoV-2 strain that is highly divergent and more transmissible and/or more virulent and/or capable of escaping the protection conferred by current vaccines and/or drugs becomes ever more probable with every new infection. Such variants might arise either via point mutations and/or insertions/deletions and/or recombination with other circulating SARS-CoV-2 lineages (Figure 1A,B) [75]. The emergence of the highly infectious Alpha, followed by a more infectious Delta, and now by an even more infectious Omicron variant is not simply the addition of a single amino-acid change, but several other correlated mutations also need to occur to maintain the structural integrity of the virus (including that of its genome) or to increase the expression of interferon antagonist accessory ORFs [25,87,88,91,186,187,188]. Furthermore, it is conceivable that a Delta–Omicron recombinant arises within the next few months, during which both variants will be circulating. Even before the emergence of Omicron, experts in the field, vaccine developers, and policymakers were already preparing contingency plans for the possible emergence of a vaccine-escape and/or more lethal variants. The relatively high mutation rate of the Spike gene [68] and the observation of insertions within the (computationally predicted) epitopes of spike [87] also favor this scenario. In addition, 3D structural simulations of spike-trimer single and double mutants at the RBD show that several mutations may give rise to even more stable structures [189]. Deep mutational scanning of the SARS-CoV-2 spike RBD revealed that, although many mutations are deleterious, a considerable number of them are either well tolerated or actually enhance binding to the ACE2 receptor [190]. The emergence of the A.30 variant and its cell entry properties [191] shows that new strains may not only evade current spike-targeted vaccines but also evolve a preference for other cell types that would promote extrapulmonary spread. Worryingly, new reservoirs of SARS-CoV-2 could be established in farmed or wild animals (including rodents [26]) and lead to the emergence of new strains with properties that would allow spill-back to humans as studies in minks have already shown [49,50].

### 16.3. Scenario 3: Intratypic Recombinations between SARS-CoV-2 and Other Sarbecoviruses

In a third less probable but still feasible scenario (Figure 1C), a SARS-CoV-2-infected person could pass the virus to an animal infected with another closely related CoV from the SARS-CoV-2 lineage, and the two viruses then recombine. By these means, an even more divergent SARS-CoV-2 strain could emerge that escapes protection elicited by current vaccines. In a variation of this scenario (Figure 1D), a SARS-CoV-2-infected person passes the virus to an animal infected with another closely related CoV from the SARS-CoV-1 lineage and the two viruses recombine. In this case, given the modular recombination potential of the spike region, a new Sarbecovirus may emerge that combines the high infectivity of SARS-CoV-2 (by acting as donor of, for example, the Omicron spike) with the high mortality rates (~10%) of SARS-CoV-1 infections. Alternatively, an accessory ORF from the SARS-CoV-1 lineage replaces via homologous recombination its ortholog in SARS-CoV-2, and the recombinant virus displays altered properties. Accessory ORF orthologs among different sarbecoviruses (i.e., ORF3a of SARS-CoV-1 and SARS-CoV-2) have been shown to use different strategies to induce apoptosis [61] with important implications for the asymptomatic spread of the virus. The feasibility of this scenario is evidenced by recent analyses of the role of recombination in *Sarbecovirus* evolution [52,76,111].

### 16.4. Scenario 4: Intertypic Recombination between SARS-CoV-2 and Viruses from Other Beta-CoV Subgenera

The spike region of a highly infectious SARS-CoV-2 passes (via modular intertypic recombination) to an animal or human CoV of another subgenus within the Beta-genus CoVs (Figure 1E) and undergoes rapid divergence due to the fact of adaptation. This might happen with a MERS or MERS-like virus obtaining the highly infectious SARS-CoV-2 spike. MERS-CoV has low infectivity but very high mortality (~30%). A recombinant of this type would be catastrophic unless rapid vaccine development and distribution occurs across the entire world. Another variation of this scenario is for an as yet undiscovered Beta-CoV subgenus to recombine with SARS-CoV-2. Although improbable, this scenario is not infeasible as bioinformatics analyses have shown [52,126], and this emphasizes the need to develop pan-coronavirus vaccines.

### 16.5. Scenario 5: Accessory ORF Acquisition by Non-Homologous Recombination of SARS-CoV-2 with Other Coronaviruses or Even Other Viruses/Hosts or Even via De Novo Gene Birth

A SARS-CoV-2 genome acquires (via non-homologous recombination) accessory ORFs from gene duplication, (via horizontal gene transfer) from other CoVs, viruses, or hosts, or even by de novo gene birth; as a result, its molecular biology changes (Figure 1F). Examples of accessory ORF acquisition events by the above evolutionary processes have already been observed in Beta-genus CoVs [52,63,144,145,192,193]. It is not yet clear what would be the implications if an accessory ORF from the SARS-CoV-1 lineage were to be introduced via non-homologous recombination into SARS-CoV-2. Finally, there are no current data that support a scenario of a homologous recombination event between SARS-CoV-2 and CoVs from other genera [52].

## 17. Conclusions—Implications for Vaccination Policies

Several studies have shown that vaccination does protect people against infection in the short term and against severe disease and death in the long term [194]. It also lowers the chance of vaccinated people infecting other people, even if a breakthrough infection occurs [194]. In addition, simulations have shown that vaccination reduces the probability of the emergence of a vaccine-resistant strain, but only if transmission control measures are maintained throughout the vaccination campaign [195]. People in the developing world frequently come into contact with wild animals that are reservoirs for many CoVs; they also have significantly lower access to current vaccines [196]. The human–animal interface is a serious source of concern for spill-over events [197]. Furthermore, in some developing countries, the number of immunosuppressed patients (e.g., due to HIV) is very high. Prolonged viral replication occurs in immune-compromised/suppressed patients and can lead to the emergence of more infectious and even vaccine-evading variants of concern [79,198]. Thus, the vaccination policies of the developed world should include the timely distribution of effective vaccines in sufficient numbers to protect populations in the developing world. This would also act as an insurance against the manifestation of catastrophic scenarios concerning the future evolutionary path of SARS-CoV-2 or to ensure that no other CoV pandemics occur in the near or more distant future. In addition, the early detection of the Omicron VoC in Southern Africa, due to the fact of rigorous genomic screening, highlights the importance of developed countries supporting genome sequencing infrastructure in developing countries. In this new era of urbanization, global transport, intensive farming, and habitat destruction, close collaboration and support among all countries is imperative in the fight against this and any future pandemic. No matter what evolutionary trajectory SARS-CoV-2 follows, sociopolitical factors and international collaboration will determine the future path of the COVID-19 pandemic.

## Data Availability

Not applicable.

## Abbreviations

3CLpro	3-chymotrypsin-like protease
ACE2	angiotensin-converting enzyme 2
AOF	accessory ORF family
APN	aminopeptidase N
CCoV	canine coronavirus
CDC	Centers for Disease Control and Prevention
CoV	coronavirus
COVID-19	coronavirus disease 2019
DPP4	dipeptidyl peptidase 4
FCoV	feline coronavirus
FCS	furin cleavage site
FIPV	feline infectious peritonitis virus
GARD	genetic algorithm for recombination detection
GISAID	Global Initiative on Sharing All Influenza Data
HEL	NSP 13 C-terminal nucleoside triphosphate-binding/helicase motif
HIV	human immunodeficiency virus
IBV	infectious bronchitis virus
mABs	monoclonal antibodies
MERS	Middle East respiratory syndrome
MHV	murine hepatitis virus
N	nucleocapsid
NiRAN	nidovirus RdRp-associated nucleotidyl transferase domain
NSP	non-structural peptide
nt	nucleotide
ORF	open reading frame
PDCoV	porcine delta coronavirus
PEDV	porcine epidemic diarrhea virus
RBD	receptor-binding domain
RBM	receptor-binding motif within the RBD of spike
RDP4	recombination detection program 4
RdRp	RNA-dependent RNA polymerase
SADS	swine acute diarrhea syndrome
SARS-CoV-2	severe acute respiratory syndrome coronavirus-2
TGEV	transmissible gastroenteritis virus
TRS	transcriptional regulatory sequence
UTR	untranslated region
VoC	variant of concern
WHO	World Health Organization
ZBD	NSP 13 N-terminal cysteine-rich zinc-binding domain.
